# Community pharmacists’ views about prescription medicine co-payments and potential implications for equitable access to medicines: a critical realist interpretation

**DOI:** 10.1186/s40545-023-00673-7

**Published:** 2023-11-27

**Authors:** Elizabeth F. Peacocke, Adam Fusheini, Pauline Norris

**Affiliations:** 1 Norwegian Institute of Public Health, Skøyen, P.O. Box 222, 0213 Oslo, Norway; 2 Department of Preventive and Social Medicine, University of Otago, Dunedin, New Zealand; 3 Va’a o Tautai, Centre for Pacific Health, University of Otago, Dunedin, New Zealand

**Keywords:** Community pharmacy services, Co-payments, Deductibles, Out-of-pocket, Social pharmacy, Critical realism

## Abstract

**Background:**

In many countries the community pharmacist’s role includes collecting prescription medicine co-payments at the point of dispensing. This is a context which can provide unique insights into individuals’ access to prescription medicines, as interactions with service users about out-of-pocket (OOP) expenses that may negatively affect a pharmacist’s patient counselling role. Prior research has identified that OOP expenses for prescription medicines led to decreased treatment adherence. This study aims to understand the role of community pharmacists in the collection of co-payments for prescription medicines in one region of Aotearoa New Zealand, and the possible implications for equitable access to medicines.

**Methods:**

This is a qualitative study using a case study research design. Data were collected through focus groups, individual interviews, and an electronic survey. Using a critical realist approach in thematic analysis, findings were categorised as Causal tendencies (the things that cause the events); Events (the things that community pharmacists experience); and Experiences (the perceptions and feelings of individual participants).

**Results:**

Our analysis finds that the current profession of community pharmacy in Aotearoa New Zealand, is under strain. The results suggest that broader government policies, such as the pharmacist’s role in delivering essential health services, the fairness of standard prescription co-payments, and the role of community pharmacists as gatekeepers, have a significant influence on the profession. In addition, the study found that individual community pharmacists have a unique position in the co-payment process, face power imbalances within their role, and the study indicates evidence of value judgements towards service users.

**Conclusions:**

This study is exploratory; however, its examination of the policy of prescription medicine co-payments from the perspective of community pharmacists, who play a vital role in both dispensing medicines and collecting prescription medicine co-payments, is novel. Despite prescription medicine co-payments being a routine part of pharmacists’ role in many countries, it is a topic where there is limited published peer-reviewed literature. The study adds to existing evidence that funding models influence community pharmacists’ role. In addition, this study identified value judgements about service users in relation to prescription medicine co-payments which may influence service users’ health-seeking behaviour. In this setting, limited representation of at-risk populations in the community pharmacy profession may be a factor that negatively influence interactions between pharmacists and service users.

## Highlights

- This study is novel as examines the policy of prescription medicine co-payments from the perspective of community pharmacists, a critical yet overlooked profession in discussions about out-of-pocket costs and implications for equitable access to medicines.
- Findings indicate that the implementation of the government financial risk protection for out-of-pocket payments for medicines in Aotearoa New Zealand, the Prescription Subsidy Scheme, may be inadequately implemented and lead to people missing out on further discount to subsidised medicines.
- The study identified value judgements by community pharmacists which may impact on their counselling role, which may influence service users’ health-seeking behaviour.

## Background

Medicines are the most common health intervention for preventing and managing illness and conditions [1, 2]. Community pharmacists are the main dispensers of medicines for primary health care in many countries [3]. In Aotearoa New Zealand (from here on referred to as Aotearoa), collecting co-payments is a routine aspect of the dispensing process. Health systems commonly use prescription medicine co-payments as a cost-sharing tool and policy, between governments and users, to support cost-containment from a health system perspective [4, 5] and to deter the over-use of medicines [6]. Across settings, increasing cost-sharing leads to decreases in treatment adherence [7–10].

### Out-of-pocket payments and implications for equitable access to medicines

Out-of-pocket (OOP) payments expose individuals living with chronic conditions to a greater risk of financial hardship due to the association between the quantity and frequency of prescribed medicines necessary to manage their condition [11, 12]. OOP payments for health services can promote inequity, particularly if they are implemented universally [13, 14], and are considered to be an ineffective rationing instrument [14]. It is for these reasons that OOP payments challenge a health system’s ability to support *equity in health*, defined as the absence of unfair and avoidable or remediable differences in health among population groups defined socially, economically, demographically or geographically [15].

### Medicine policy in Aotearoa: publicly subsidised pharmaceuticals with financial protection from catastrophic OOP expenses

The health system of Aotearoa is largely publicly funded, with most prescription medicines funded with tax revenue by *Te Whatu Ora* (Health New Zealand) [16]. Government funding subsidises the cost of the medicines for individuals, and health service users are required to pay a capped payment for medicines at collection from the community pharmacy. At the time of completing this research, Aotearoa had a universally applied standard prescription medicine co-payment, in most cases this was NZ$5.00 (USD$3) for fully subsidised medicines, and $15.00 (USD$9) for medicines prescribed by specialists [17]. The strategy to prevent service users from catastrophic expenditure is the Prescription Subsidy Scheme, a cap of 20 items (NZD$100); however,  medicine collection may not be evenly distributed across the 12-month period [17]. People prescribed large quantities of medicines were required to pay the NZD$100 early in the 12-month period, compared with those where prescriptions are spread across the calendar year. Despite this being considered low by international standards, the results of a recently conducted randomised control trail investigating whether exempting people from the $5 prescription charge in Aotearoa reduces hospital use, found that those in the intervention group were significantly less likely to be hospitalised during the study year than those in the control group [18]. The policy environment for this issue is very active, and the NZD$5 co-payment was removed for all prescription medicines from 1 July 2023 (with charges for medicines prescribed by a specialist remaining) [17]. However, with the election of a new government in October 2023, this policy is being considered to be repealed, the outcome of which was still unknown at the time of this publication [19].

### Impact of the collection of prescription medicine co-payments by the community pharmacy and the role of the community pharmacist

Patient counselling is an important and influential part of the practice of community pharmacy [20–22]. In particular, giving advice, education, and the development of personal relationships can support improved health outcomes for service users [20, 22]. The interaction between the service user and a community pharmacist should be a clinical task, where advice about medicines can be given [22]. However, when the price of the medicine is raised at point of dispensing, community pharmacists may influence service users’ decisions about the prioritisation, or ‘cherry picking’ of medicines [23]. Encouragement from pharmacists’ over generic selection indicates they can have an influential role in the interaction with service users [24, 25]. Several researchers have suggested that counselling by community pharmacists related to co-payments could be a factor that affects the collection of prescriptions [21, 23, 26].

The profession of pharmacy has both professional and business orientations [27], and the type of reimbursement structure for community pharmacy can have a direct impact on practice patterns [28, 29]. Dispensing payments are reported to influence pharmacists’ behaviour [30, 31], for example, increasing the quantity of prescriptions dispensed [31]. Several studies from Aotearoa have presented the impact of prescription co-payments on peoples’ understanding of, and access to, prescription medicines as a system that is inequitable, disproportionately affecting at-risk communities [9, 10, 23, 32, 33]. Building on a previous study that investigated the effect of increased prescription charges on community pharmacies [26], this study is novel as it examines the policy of prescription medicine co-payments from the perspective of community pharmacists. The study aims to understand the role of community pharmacists in the co-payment process for prescription medicines in one region of Aotearoa and the implications this may have for equity.

## Methods

This is an empirical inquiry investigating a contemporary event using case study design [34]. Qualitative research methods were used, studying prescription medicine co-payment processes among community pharmacists in their natural setting [35]. Qualitative research was considered appropriate for this study as the objective is to understand the issue and process, rather than to look for causal relationships [36].

### Conceptual framework and theoretical underpinnings

The theoretical underpinnings of this study are influenced by critical realism, which uses a realist methodology, where the researcher considers the interplay between contextual elements and mechanisms of action [37, 38]. The critical realist approach examines how interventions—for this study, prescription medicine co-payments—are influenced by particular contexts rather than ‘universal truths’ [37, 38]. Critical realism emphasises that reality exists independent of individual perceptions, and therefore, it may not be directly observable or measurable [39]. A critical realist philosophy influences the theory by describing the phenomena as a reality (ontological realism). At the same time, the epistemology is aligned with the subjectivist position of interpretivism, concentrating on how we perceive, construct, interpret and invent our experiences [39, 40]. Critical realism examines current phenomena to unveil the ﻿‘real state of affairs’, including a critical analysis of power relationships [40]. To explore power relationships in this study, data were collected to explore the differing perspectives between pharmacy owners and employees.

### Study context

Community pharmacies are privately owned and operated in Aotearoa. Most pharmacies’ income and operations include a mix of retail sales and dispensing of prescription medicines [41]. They are contracted by *Te Whatu Ora* to provide a fee-for-service dispensing function to purchase and then dispense prescribed medicines, which they then reclaim the cost of as a reimbursement from the New Zealand Ministry of Health (MoH) [16, 42]. In addition, they deliver specific services, including long-term conditions management, and methadone programmes, amongst others [41]. Community pharmacies purchase medicines from wholesalers, which they then reclaim from the MoH once the medicine has been dispensed. At the time of conducting this study, the value of the co-payment was deducted from the cost of the pharmaceutical when this is reclaimed from the MoH [42].

### Participant selection

The location of this case study was the Bay of Plenty (BOP) region, Aotearoa (population of 259,090 people in 2020/21), the region has more Māori (Aotearoa’s indigenous population), more people living in income quintiles 4 and 5 (the two most deprived quintiles) than the national average [43], and there are also large rural pockets in the region. Participation selection was purposive, participants needed to be community pharmacists familiar with administering co-payments and the relevant claims processes with MoH. Attempts were made to recruit participants from a mix of ages, gender, and ethnicities, prioritising community pharmacists who worked in areas of high deprivation,^1^ and community pharmacists who identified as Māori.

### Study design

The study included two focus group discussions, six individual interviews, and a short electronic survey to participants about the processes they follow in managing co-payment processes.

### Data collection

The interviews, focus groups and survey were completed between March and April 2022. An electronic survey of 17 questions developed in Qualtrics [44] was administered to collect information about what community pharmacists have to do in relation to standard prescription medicine co-payment processes. It was emailed or messaged to all participants following the focus group or interview (S1). The survey was a combination of closed, single or multiple select nominal questions.

Focus groups, and interviews followed a typical case study research design. The topic guide (S2) was designed to facilitate deductive, abductive, retroductive, and inductive reasoning and analysis. Open-ended questions were included to enable the identification of theoretical concepts unknown to the inquirer, that could be discussed by study participants. Two focus group discussions were held, one with community pharmacy owners, one with employees (none of the owners had employees in the focus group), and individual interviews (one interviewee was from the same pharmacy as an owner in the focus group). A box of chocolates was offered to participants following the interviews as a small gift to acknowledge the use of their time.

### Data analysis

Data collected from the survey were used to triangulate findings from the focus groups and interviews. Simple statistical analysis was performed and are presented in narrative form.

Focus groups and individual interviews were recorded through Zoom and verbatim transcripts were drafted, aided by the software otter.ai [45]. Guided by Braun and Clarke’s guide for thematic analysis (2021), semantic and latent coding in the analysis of discussion data was used to examine patterns of explicit content in the data. NVivo was used to document the codes (released in March 2020) [39]. Thematic analysis was informed by a critical realism [46, 47], where we explored the representation of the empirical, actual and real in the data [38, 46]. We studied the data to produce evidence-informed theories about the interactions between co-payments (intervention mechanisms) and the contexts in which they are implemented [38, 47]. The coding process was initially inductive as media coverage indicated that the community pharmacy sector in Aotearoa was under stress; with a deductive approach also used as we were open to what the participants reported about the collection of OOP payments. In addition, we wanted to apply a critical realist analytic framework that would use both abduction (using theories to make sense of the data) [39] and retroduction (the activity of theorizing and testing for hidden causal mechanisms responsible for manifesting the empirical, observable world) [38, 47]. The first author (EP) conducted the first stages of analysis. To support rigor, validity, and trustworthiness, PN and AF contributed to the development of themes through discussion, offering their professional interpretation of the data.

### Reflexivity

The authors are researchers in health systems, health policy and social pharmacy. The lead researcher (EP) has professional experiences in government health service regulation, planning and funding, with insights into government contracting with health providers. Throughout the study design, data collection and analysis, journaling and note-taking was used to expose pre-conceived ideas about the research. Recording decisions supported the reflexivity throughout the research [39]. This reflexivity is included in the analysis.

### Reporting

We report the findings using a framework of critical realism suggested by Wiltshire and Ronkainen (2021) that captures the observed (empirical), unobserved (actual), and unobservable (real) [46], while remaining grounded in the participants’ accounts and the realities in which the participants are situated [46, 47]. The unobservable themes are reported as the findings, presented as **causal tendencies**—the things that cause the events, **events**—the things that are experienced by community pharmacists and **experiences**—the perceptions and feelings of individual participants (verbatim quotes from the data) [47]. The findings include a critical position in the associated burden on these practitioners and the potential influence that this may have on equitable access to medicines.

### Ethical approval

The University of Otago Human Ethics Committee granted a Category B ethical approval, which can be approved by a Head of Department as the study was focused on individuals professional roles, and did not collect personal information. All participants were informed about the purpose of the research and signed informed consent to participate in the study.

## Results

One focus group of community pharmacy owners had four participants (two female, two male). The other included community pharmacy employees with three participants (two female, one male). In addition, individual semi-structured interviews were held with two owners (one female, one male), and four employees (three female, one male). All participants were professional community pharmacists, located and working in the BOP DHB region. Two pharmacists were employed at the same pharmacy. The ethnicity of participants was self-selected, and reflects the diverse population of the region. Geographically, nearly half of the participants (*n* = 6) were based on areas of high deprivation, and slightly fewer (*n* = 4) were located in rural or remote areas (S3).

### Part one: results from survey

#### Impact of collecting co-payments on the role of community pharmacists

We collected information on the requirements and mitigations that community pharmacists follow in administering medicine co-payments in Aotearoa. This study identified a convoluted process that community pharmacists follow from the point that a prescription is delivered, through dispensing, collection of co-payments and then government reimbursement for the cost of the pharmaceutical [48]. The responsibility for collection of co-payments sits with the community pharmacist, if the co-payment is not collected from the service user, then this is a loss to the business. All survey respondents (*n* = 9, of 13 participants) agreed that they had experienced having to support service users who cannot afford co-payments. Results indicate that it is a significant part of routine work. Participants emphasised the steps that they went through to manage the service user not paying. These included: offering items on account or credit, giving small quantities of medicines for a temporary fix, waiving the co-payment, service users cherry-picking their medicines, or the service user leaving without collecting prescription medicine(s) [48] (Table 1).

**Table 1:** Participant demographics

Gender^§^	Female	8
	Male	5
	Total	**13**
Employment status^§^	Employee	7
	Owner	6
	Total	**13**
Years of professional experience as a pharmacist^*^	< 6 years	1
	6–10	1
	11–15	2
	16–20	2
	21 +	3
	Unknown (did not complete survey)	4
	Total	**13**
Ethnicity (self-selected) (based on New Zealand Census classifications)^*^	New Zealand European	5
	Unknown (did not complete survey)	4
	Māori	2
	Chinese	2
	Samoan	0
	Cook Islands Māori	0
	Tongan	0
	Niuean	0
	Other, e.g., Dutch, Japanese, Tokelauan	0
	Preferred not to say	0
	Total	**13**
Pharmacy type^§^	Independent	9
	Banner	3
	N/A (Locum)	1
	Total	**13**
Pharmacy location (could select more than one option)^*^	Located in an area of high deprivation	6
	Located in a rural or remote area	4
	Neither applicable	6
	Total	**16**

Data collected by researcher^§^ or in the survey*

### Part two: results from focus groups and individual interviews

Results pertain to the current practice of community pharmacy, government policy, and individual pharmacists. Influenced by Fryer’s critical realist approach to thematic analysis [47], Table 2 presents possible causal explanations of the pharmacists role in managing co-payments that may influence equitable access to prescription medicines.

**Table 2:** Summary of study themes that describe how the implementation of the standard prescription medicine co-payment may influence equitable access to prescription medicines

Causal tendencies *The things that produce the events *[47]	Events *The things that are perceived by agents (community pharmacists) *[47]	Experiences *The perceptions of things by agent (community pharmacist) *[47]
**The influence of working as community-minded professionals**		
A sense of community, and the desire to help people provide meaning in the profession of pharmacy	Individuals who practice community pharmacy are committed to ensuring that service users are not negatively affected by standard prescription medicine co-payments	*"I believe it should be done on a whanau [family]-centric patient model, obviously, and so reducing costs for them when necessary. As opposed to imposing more costs… I think as a sector, …we need to highlight to the Ministry and the government, that that does not work. Right. Those are what cause inequities in the system. And unless we’re doing things collectively, the same way, it’s never gonna work for anybody..”—Employee, Individual interview*
	Community pharmacists are often conflicted by the idea that service users require medicines but may not be able to afford them	*“So, if people are coming in with a prescription for antibiotics and don’t have the capacity to pay or anything like that, then we would tend to find a solution to support them… Most of the time… I would tend to just issue the whole script and just say ‘Look, when you can, when you can come back and pay for it, then that’s fine.’”—Employee, Individual interview*
**A profession under strain**		
The practice of community pharmacy is exposed to demands of a small private business and the associated instability of income, affecting the role of the community pharmacist	Due to the current context of community pharmacy and the perception of increasing demands, community pharmacists feel that the profession is struggling in the environment	*“…there are so many demands on pharmacy at the moment. They’ve given us all these extra tasks we can do, and we get paid for them. But it’s always an opportunity cost and if you’re going to … do this or do that, you’ve got your… other work is piling up.”—Employee, Individual interview*
		*“…at the moment, pharmacies are mainly competing in the sector, and how much cheaper they can make their services, rather than how well they provide their services… The services that we don’t get payment for, it’s really difficult to justify from a business perspective…”—Employee, Focus group*
	Community pharmacies in Aotearoa have been disadvantaged by the introduction of discount pharmacies	*“We do everything, we go further, we deliver, we do all those sort of things. We need the $5 currently to afford to do that, if we lose that. It’s a slow, agonising death. And we’ve already seen, in my other role, it’s pharmacies going under, more and more every year, having to close the doors because of this one thing…”—Owner, Focus group* *“It should be the same playing fields, everybody, you can’t let some give off, and some can’t. It’s bad management on behalf of the profession, yeah. In my opinion, yep.”—Employee, Individual interview*
	Some community pharmacists are not willing to discount the standard prescription medicine co-payment when participants cannot pay, meaning that some service users will not access their medicines	*“Honestly, sometimes I do tell them to go to Chemist Warehouse [a discount pharmacy], just because like, this, I know they can’t afford it. And I know that Chemist Warehouse does it free for them. And we, because we’re private, we can’t give prescriptions for free and maintain, you know, like a business by just giving medications for free. And I know that Chemist Warehouse can absorb that cost. And some circumstances I do, say like, ‘Hey, Countdown’s down the road, they are free of charge. You can like walk there and try and get your prescription from them instead.’ Because it’s, we’re losing the customer. But if we take the cost, we’re losing actual money. You know, we’re paying that co-payment that the patient should be paying to the government. So, like, it’s a shame, but it is the only real alternative that you can keep, err, without losing money.”—Employee, Focus group*
**A sector that is committed to delivering an essential health service**		
The community pharmacy sector perceives itself as part of the health system, believing that they better adhere to the service users’ needs, which may result in a higher standard of care at the banner or independent community pharmacies, compared to discount pharmacies	Some community pharmacists might be providing a higher quality service than discount pharmacies	*"… some people may take their medicines really, really, really well. And the fact that and they’re very adherent to the medication regime, but they just can’t afford that cost… conversely, you have other patients… that may not take their medicines well at all, but they will always go to the place that it’s free. And that doesn’t necessarily mean Chemist Warehouse and Countdown [discount pharmacies] provide poor services, it just means that they go there for… free prescriptions, but then mainly, but if Chemist Warehouse and staff are really busy, they may not have the time to fully counsel the patient, you know, for for the medications and the fact that whether they’re taking up properly or not, and that may have some flow-on effects later on. Like they may not, you know, they may be taking the medicines incorrectly for a really long time, but because it’s free, they’re just continuously going back"—Employee, Focus group*
	Some discount pharmacies are not fulfilling contractual requirements by choosing not to fill all prescription items	*“… you’ll find that these discounters will quite often turn people away and say that they can’t do it because they don’t have the raw materials to do it or for whatever reason, and you will end up having a prescription that’s stamped with, you, so you know, that they’ve been elsewhere… it’s, it’s frustrating because they’re not fulfilling their contract with their DHBs… it’s meant to be a one-stop shop.”—Owner, Individual interview*
**The fairness of co-payments for community pharmacy and service users**		
The implementation of the government co-payment policy has had unintended consequences, and may undermine original policy goals	There are inconsistencies between government policy intentions of the standard prescription medicine co-payment and implementation	*"Once again, and during and around COVID times we’ve seen that things that we’ve constantly asked for, which is a direct payment from Work and Income [social services] to pay for medication need and for medical expenses, which has always been, you know, denied, have been able to be implemented. You’re in COVID with certain groups getting access to directly charge Work and Income for families who cannot afford to get their medication or food. So currently, the system that we have doesn’t really help facilitate any of the issues that we have around co-payment other than those people going outside the regional area to access free scripts…. It’s quite a challenge."—Owner, Individual interview*
	Community pharmacists are subordinate to the demands the government has in relation to standard prescription medicine co-payments and related requirements	*"But yeah, there has to be a cut off somewhere. It won’t go away. I don’t, I don’t think it will, the government will utilise the fact that the pharmacy sector will be allowed to be able to absorb that debt and make it the sector’s problem to deal with it rather than the government’s problem to deal with it."—Owner, Individual interview*
	Government departments are influencing equitable access to medicines by encouraging service users to collect medicines from discount pharmacies	*"…and then the government’s quite happy for us to collect the $5 charge, but they’re now quite happy for, in a number of scenarios to direct patients to the people that give free, don’t charge co-payments…”—Owner, Focus group*
	Community pharmacists often understand that the standard prescription medicine co-payment system is regressive and inequitable, disproportionately affecting those with co-morbidities and high medicine usage	*"…someone that’s, you know, only taking the medications all in the morning or something, they would only pay $5 per month so that’s quite tricky as well because… they can’t kind of control, like, what medications they get put on, and how often they’ll need to take them… So yeah, it’s a bit tricky in that regard as well."—Employee, Individual interview*
**Community pharmacists’ role as gatekeepers and government policy enforcers**		
The role of health professionals as gatekeepers and policy enforcers of health entitlements may contribute to inequitable implementation	Some service users may not receive a further subsidy to the standard prescription medicine co-payment because the sole responsibility to check eligibility sits with the community pharmacist	*“So quite often, and it happens quite regularly. You’ll, you’ll you’ll charge them… for the scripts, the $5, and then they’ll say, ‘Oh, my wife gets prescriptions at another pharmacy.’ And so we’ve just gotta go backtrack and start all over again and get the right price and do a bit of background work on that. So that’s challenging and takes up time, and resources.”—Owner, Individual interview*
	The practice of community pharmacy often requires community pharmacists to implement and enforce the government policy of the standard prescription medicine co-payment	*"…you notice it more when the pharmacies sort of came out that weren’t charging for scripts. You know, like, you would kind of get questioned a lot like, ‘…why are you charging this to me? And why are they not? And how…’ you know, ‘how can they do it and you can’t?’ Kind of thing. Like, I find it quite tricky to explain it to patients as to why you know, that it’s more like a tax and that kind of thing. And that, you know, if you don’t pay it, then we pay it for you…"—Employee, Individual interview*
**Power imbalances exist within the professional role**		
The dynamic of employer and employee, whereby pharmacy owners hold more power, can limit an employee community pharmacist’s ability to support access to medicines when service users cannot pay	Individual community pharmacists do not feel that they have the authority to ensure that service users receive access to medicines if they cannot pay standard prescription medicine co-payments	*“So, it can be a barrier for some, but fortunately, for our regular patients, particularly our patients who are on, you know, anywhere between five to 12 medicines, they will typically have an account. So, they can pay that off slowly, which I think makes a huge difference in terms of access, and health outcomes overall. But it is, you know, at the discretion of my boss… and lately, they’re wanting to cut, or stop, any new accounts… So it is, yeah, that’s a business loss in a sense or a business decision that they’ve made in the past for better health outcomes. But yeah, it’s a balancing act.”—Employee, Individual interview*
Community pharmacists are aware of the power they hold over service users in accessing medicines equitably	Community pharmacists, like other health professionals, play an influential role in access to medicines	*“I’m not ever going to judge where people spend their money. That’s not my job. But if it means that they can’t get their medicines that day because they don’t have any left or they don’t get paid till the next day, then it’s my role to ensure that they have access to medicines”—Employee, Individual interview*
**Community pharmacists’ unique place in the co-payment process**		
Health professionals’ position in the health system may influence their perspective and understanding of a patient’s needs	Some health professionals are making assumptions on behalf of service users, influencing patient autonomy by directing service users towards discount pharmacies	*“But a lot of people are being led towards them [discount pharmacies] by health professionals that aren’t pharmacists, like when I was working in the hospital… a lot of the time the doctors just send them to a free chemist rather than their regular ones, just because it is a free service. And they think it’s going to benefit them without realising that no, they actually have an existing relationship with … a pharmacy and all this stuff.”* *- Employee, Focus group*
	Community pharmacists recognise that standard prescription medicine co-payments prevent people from accessing prescription medicines	*“But for co-payments, I reckon sometimes $5 is a bit too dear for quite a large, you know, particularly in Māori and Pacific or people that are, that just don’t make enough money simply $5, or let’s say you need to be on medications, like a bunch of blood pressure, you know, diabetes meds, or some gout meds or something like that.”—Employee, Focus group*
**Evidence of value judgements towards service users**		
Health professionals’ beliefs of individualism and personal responsibility may influence interactions with service users	Community pharmacists sometimes make value judgements about what they believe to be choices that service users make in relation to collecting, or not collecting, prescription medicines	*“There is the option to take a copy of the receipt and go to WINZ [Work and Income New Zealand] and get a card allocated to the amount of the prescription, and WINZ will do that. We’ve got a WINZ office pretty much straight across the street. That is an option but it takes time, of course, so they have to swallow their pride sometimes and go and do that. But that is an option…. But as I say, they’ve got to have the will to do it, in the time.”—Owner, Focus group*
		*“…they’re so willing to spend money on everything else to do with their health, but just not, you know, they’ll spend $20 on a vitamin, and then they won’t want to spend $5 on their blood pressure tablets. A lot of people genuinely can’t afford it. But a lot of the time, I feel like there’s no real understanding of like how, you know, the system is actually benefiting them in a lot of ways”—Employee, Focus group*
	Some community pharmacists believe the moral hazard argument, that if people contribute towards the cost of their healthcare then they value it more	*“You’re probably gonna end up with a huge amount of wastage because, people, if they got everything for nothing, they don’t attach any value to it. So, by paying a little bit of the $5 each time, they, they, attach a slight bit of value to it.”—Employee, Individual interview*

#### The influence of working as community-minded professionals

We identified that Individuals who practice community pharmacy are committed to ensuring that service users are not negatively affected by standard prescription medicine co-payments. They reported feeling conflicted by the idea that service users require medicines but may not be able to afford them, and an ‘ethical dilemma’ if they needed to withhold dispensing due to lack of payment. In situations, where they felt that a service user’s access to their medicines was most important, participants described an internal conflict acknowledging that not collecting the co-payment would come at a financial loss to the pharmacy.

“So, if people are coming in with a prescription for antibiotics and don’t have the capacity to pay or anything like that, then we would tend to find a solution to support them…”—Employee, Individual interview (Table 2)

#### A profession under strain

Participants indicated the community pharmacy sector is currently struggling, one participant expressed this in relation to the strain of increased competitive behaviour within the sector. A unique factor currently affecting community pharmacists in Aotearoa is the introduction of discount pharmacies which waive prescription medicines co-payments, offering medicines for free. Most participants reported that it was affecting how the pharmacy operates.

### A sector that is committed to delivering an essential health service

Participants reported feeling that they provide a higher standard of service than discount pharmacies. Participants implied that this was due to the community pharmacy sector perceiving itself as part of the health system, with several participants referring to a higher standard of care, or implying that they follow their clinical responsibilities related to service users’ needs better than discount pharmacies. Some participants reported that some discount pharmacies are not fulfilling contractual requirements by being selective about the prescription items they dispense.

“... you’ll find that these discounters will quite often turn people away and say that they can’t do it because they don’t have the raw materials to do it or for whatever reason…”—Owner, Individual interview (Table 2)

### The fairness of co-payments for community pharmacy and service users

The prescription medicine co-payment policy is intended to contain the cost of medicines to the service user [49]. Findings from this study indicate there are inconsistencies between the intention of this policy and how it’s implemented. Community pharmacists reported that the medicine co-payment system that is based on a total payment cap is inequitable, because the policy is implemented unilaterally, and the burden of the co-payment falls disproportionately on certain groups, e.g., people with co-morbidities who may be prescribed a lot of medicines at once.

### Community pharmacists’ role as gatekeepers and government policy enforcers

Most participants in this study reported that they were involuntarily collecting the standard prescription medicine co-payment on behalf of the government.

“...you notice it more when the pharmacies sort of came out that weren’t charging for scripts. You know, like, you would kind of get questioned a lot like, ‘…why are you charging this to me? And why are they not?...’”—Employee, Individual interview (Table 2)

Most participants in this study recognise that access to the financial risk protection through the Prescription Subsidy Scheme, rests with them as pharmacists in terms of checking the service user’s status and eligibility. This can sometimes lead to people missing out on their entitlements. As confirmed by one participant, who highlighted that a person’s eligibility for the financial risk protection may be mistakenly overlooked by the community pharmacist:

“So quite often… you’ll charge them… for the scripts, the $5, and then they’ll say, ‘Oh, my wife gets prescriptions at another pharmacy.’ ”—Owner, Individual interview (Table 2)

### Power imbalances exist within the professional role

The evidence presented thus far indicates that community pharmacists feel powerless to the whim of government policy. Within the pharmacy itself, community pharmacist employees (as opposed to pharmacy owners) indicated that they felt limited in their ability to support access to medicines in situations when service users cannot pay. Many participants mentioned deferring to the owner or manager of the pharmacy to approve setting up credit or waiving charges for service users.

Most participants in this study reported their role in enabling a service user to leave the pharmacy with or without the medicine(s) they had been prescribed, indicating awareness of the power they hold over service users in accessing medicines equitably. One participant indicated that they have experienced situations, where the tone of the interaction between the service user and community pharmacist could lead to inequitable outcomes.

“I have had to step in [on other people having conversations], because it’s got quite fiery or, you know, they [the service user] are about to walk out or feel that they haven’t been listened to maybe, or maybe embarrassed.”—Employee, Individual interview (Table 2)

### Community pharmacists’ unique place in the co-payment processes

Community pharmacists’ position within the health system offers them privileged insight into medicine co-payments. Most participants expressed the view that the co-payment is an impediment for those economically worse off who tend to have worse health outcomes and may face negative consequences if they do not access their medicines.

“…it generally seems to be those that can’t afford their medications that sort of have the worse health outcomes.”—Employee, Individual interview (Table 2)

### Value judgements towards service users

We observed different approaches to service users in individual interview—where participants who identified as Māori offered a different perspective on prescription medicine co-payments, compared to other study participants, particularly in relation to explaining the rationale and process of co-payments to service users. Some community pharmacists made value judgements about what they believe to be choices that service users make in relation to collecting, or not collecting prescription medicines. Participants implied that they thought that some service users willingly chose not to collect prescriptions, indicating that they disagreed with service users’ choices.

“… they’re so willing to spend money on everything else to do with their health… and then they won’t want to spend $5 on their blood pressure tablets”.—Employee, Focus group (Table 2)

Finally, many community pharmacists reported their belief that if people contribute towards the cost of their health care then they value it more, also known as the moral hazard argument [27, 50]. Most participants mentioned that free medicines could mean that there would be more wastage in medicine dispensing.

## Discussion

This explorative qualitative study aimed to understand the role of community pharmacists in prescription medicine co-payments and the implications this may have for equitable access to medicines in one region of Aotearoa. Data were collected through focus groups, individual interviews, and survey. Thematic analysis was influenced by critical realism, exploring how community pharmacists’ role in prescription medicine co-payments is influenced by particular contexts, rather than something that is universally true [38]. The possible causes and related findings were then presented using a framework of Causal tendencies, Events and Experiences (Table 2). Our analysis found that the current profession of community pharmacy is under strain. The results suggest that broader government policies, such the pharmacist’s role in delivering essential health services, the fairness of standard prescription co-payments, and the role of community pharmacists as gatekeepers, have a significant influence on the profession, which may influence service delivery. In addition, the study found that individual community pharmacists have a unique position in the co-payment process, face power imbalances within their role, and findings indicated evidence of value judgements towards service users.

This study’s participants included a mix of ages, gender, and ethnicities, prioritising community pharmacists who worked in areas of high deprivation,^2^ and Māori community pharmacists. The value judgements towards service users identified in this study, raises questions about the number of Māori health professionals in Aotearoa, a group significantly under-represented in community pharmacy compared the national population [51]. This may influence the delivery of culturally appropriate services to minority populations [52]. The third article of *Te Tiriti o Waitangi*, the founding document signed between the British Crown and some Māori, places greater obligations on the Crown to promote equity for *tangata whenua* (indigenous people) [53]. Current efforts to improve health outcomes for Māori in Aotearoa could be further promoted through culturally appropriate services, delivered by culturally competent health professionals.

The interaction between community pharmacists and service users at the point of co-payment collection is a significant aspect of the prescription medicine co-payment process. Therefore, it is an important consideration when exploring the factors that may influence service users’ decisions related to accessing medicines. The role of the community pharmacist as a counsellor has been widely reported in the literature [20–22], and an earlier study from Aotearoa has reported that community pharmacies could benefit in being more engaged in clinical counselling activities [41]. This study reports positive and negative perspectives about service users; perspectives which may also be present in counselling or interactions between the community pharmacist and service users accessing medicines. Previous studies have found that the advice, education, and personal relationship of community pharmacies can encourage improved health outcomes for service users [20, 22]. A systematic review of experiences of Māori in Aotearoa’s public health system found that Māori patients identified organisational structures and staff interactions as barriers to access, and specifically, that they were aware of negative perceptions by health professionals [54]. Health professionals’ beliefs in individualism and personal responsibility may affect interactions related to a service users’ health. In addition, the type of counselling of service users by community pharmacists could have a positive or negative impression that affects access to medicines. Referred to as bias by one recent review of Aotearoa’s health system [55], and racism by the Waitangi Tribunal [51], access to quality primary healthcare in Aotearoa is affected by both personally mediated and institutional racism [56].

The scope of this study was limited to the role of community pharmacists in prescription medicine co-payments, and, therefore, does not report examples of personally mediated racism as a finding. However, culture is known to be a determinant of health [57, 58], and previous studies have found that gaps in cultural competence can impair the delivery of health services in a culturally sensitive way. This influences patient satisfaction and adherence to treatment [59, 60]. Racism in health systems and the related effect on clinical or service delivery is associated with poorer healthcare for minority populations [54, 56, 61]. Inequitable access to medicines in Aotearoa is well-documented by government departments, and independent researchers. Shortcomings in treatment and access to medicines are reported to predominantly affect Māori, Pacific peoples, those living in high socioeconomic deprivation, those residing in rural and remote areas, and those from former refugee backgrounds [33, 62–64].

Our study revealed a complex environment of unfairness and power imbalances, where the individual community pharmacist has, both the power to withhold medicines from service users while also being powerless in the necessity to collect the co-payment. The reported power structures were divided by participants as either owner or employee community pharmacists. The study found that, generally, employees tended to express empathy towards the service user and frustration that they were unable to provide further help in circumstances where they felt that the service user needed support with co-payments. Owners expressed feeling protective of staff having to face unpleasant encounters with service users related to collecting co-payments. They also expressed concern about the long-term viability of the sector, with several mentioning looming closures of community pharmacies because of the current environment with discount pharmacies, and annoyance at having to collect co-payments on behalf of the government. Both groups (owners and employees) equally expressed concern over the cost ceiling, the Prescription Subsidy Scheme—despite seeming fair in theory—was very challenging to manage, leaving those with the greatest needs worse off.

Some participants reported that although they recognised that prescription medicine co-payments prevent some people from accessing prescription medicines, in some cases this was seen as secondary to the imperative of business viability, which outweighed service users’ need to access medicines. The ‘ethical dilemma’ of whether to withhold potentially life-saving medicines when users cannot afford standard prescription co-payments has been discussed in other studies [26, 27]. A study from Australia reported that there was a general lack of training in professional ethics in pharmacy, and that instead the best interest of the patient tended to be personally mediated through the reasoning, practical skills and personal morals of the pharmacist to manage these ethical dilemmas [27].

The influence of regulation and contracting on the role of community pharmacists has been reported as requiring them to adopt the role of ﻿‘policy enforcer’ [65]. Health professionals’ role as gatekeepers and policy enforcers of health entitlements can risk inequitable implementation for the service users. The collection of co-payments at point of dispensing in Aotearoa aligns with this. In addition, this study found that the government’s intention to cap the amount of money that service users spend on prescription medicines with the Prescription Subsidy Scheme was insufficient. At the time of conducting this study, this financial risk protection relied solely on community pharmacists accessing a specific MoH website to check the number of prescription items a service user had collected since February 1st, with no additional processes taken by government health agencies to ensure that service users received their entitlement. As mentioned by participants, it is likely that some service users or families may not receive this subsidy despite being eligible for it. The government’s decision to remove co-payments for prescription medicines from 1 July 2023 [17] is a major reform to the Prescription Subsidy Scheme. However, given the shortfall in this policy in Aotearoa, it is a warning to governments worldwide about how inadequacies related poor implementation of financial risk protections can have unintended consequences and undermine original policy goals to cap user charges.

### Strengths and limitations of study

A strength of this study is that it adds to a limited pool of international literature on the community pharmacists’ perspectives on prescription medicine co-payments. The study population is another strength as it is a representative population, including a close to even proportion of female and male participants, and diverse perspectives from minority populations, those working in rural settings, or areas of high deprivation. A purposive sampling approach was used, as the sector was under pressure with the COVID-19 pandemic during the study recruitment period. Two participants in one focus group were employees at the same pharmacy; therefore, their perspectives and experience related to the issue could be similar and be influenced by each other. The level of comfort between them could have potentially facilitated the conversation or could have also inhibited their willingness to discuss the issue. It seems unlikely that this would have influenced the overall findings of the study. There are currently unique challenges to community pharmacy in Aotearoa with the introduction of discount pharmacies and then the discontinuation of OPP, which means that the results are specific to the context of Aotearoa in 2022. As the survey was optional, only 9 out of a possible 13 responses were received. The small population means that the findings cannot be generalised to all community pharmacists in Aotearoa. Despite these limitations, some transferrable findings may be relevant to the whole sector.

### Future work plan

Further research could examine other small charges for healthcare that may affect relationships between service users and health professionals that could contribute to inequitable access to services.

## Conclusion

This exploratory study provides initial insights into the perspectives of community pharmacists about standard prescription medicine co-payments. Despite collecting co-payments being a routine part of community pharmacists’ role in many countries, it is a topic, where there is limited published peer reviewed literature. The study adds to existing evidence related to the impact of interactions between health professionals and service users, and that funding models can influence community pharmacists’ role in co-payments. The value judgements about service users in relation to prescription medicine co-payments highlighted in this study may influence service users’ health-seeking behaviour. In this setting, limited representation of at-risk populations in the community pharmacy profession may be a factor negatively influencing interactions between pharmacists and service users.

## Data Availability

To protect the participants’ privacy, the data sets used and/or analysed during the current study are not available.

## Abbreviations

BOP DHB: Bay of Plenty District Health Board
MoH: New Zealand Ministry of Health
NZD: New Zealand Dollar
OOP: Out-of-pocket
WHO: World Health Organisation

## Footnotes

1 Those Pharmacists located in a census area unit categorised as NZDep 8, 9 or 10 according to Stats NZ.

2 Those Pharmacists located in a census area unit categorised as NZDep 8, 9 or 10 according to Stats NZ.

## References

[1] National Institute for Health and Care Excellence. Medicines Optimisation: The Safe and Effective Use of Medicines to Enable the Best Possible Outcomes 2015 [cited Nov 12 2021]. Available from: https://www.nice.org.uk/guidance/ng5. 26180890

[2] World Health Organization. Monitoring the building blocks of health systems: a handbook of indicators and their measurement strategies: World Health Organization, Geneva. 2010.

[3] World Health Organization. The legal and regulatory framework for community pharmacies in the WHO European Region. 2019. Report No.: 9289054247.

[4] Barnieh L, Clement F, Harris A, Blom M, Donaldson C, Klarenbach S (2014). A systematic review of cost-sharing strategies used within publicly-funded drug plans in member countries of the organisation for economic co-operation and development. PLoS ONE.

[5] Luiza VL, Chaves LA, Silva RM, Emmerick ICM, Chaves GC, Fonseca de Araújo SC (2015). Pharmaceutical policies: effects of cap and co-payment on rational use of medicines. Cochrane Database Syst Rev.

[6] Epp J, Parkinson B, Hawse S. Health system sustainability: the pharmaceutical benefits scheme in Australia. Industry and Higher Education: Springer; 2020. p. 13–44.

[7] Gupta S, McColl MA, Guilcher SJ, Smith K (2018). Cost-related nonadherence to prescription medications in Canada: a scoping review. Patient Prefer Adherence.

[8] Ong SE, Kai Koh JJ, Toh SAES, Chia KS, Balabanova D, McKee M, et al. Assessing the influence of health systems on type 2 diabetes mellitus awareness, treatment, adherence, and control: a systematic review. PLoS ONE. 2018;13(3). 10.1371/journal.pone.0195086 PMC5875848 29596495

[9] Jatrana S, Crampton P, Norris P (2011). Ethnic differences in access to prescription medication because of cost in New Zealand. J Epidemiol Community Health.

[10] Jatrana S, Richardson K, Norris P, Crampton P (2015). Is cost-related non-collection of prescriptions associated with a reduction in health? Findings from a large-scale longitudinal study of New Zealand adults. BMJ Open.

[11] Bentur N, Gross R, Brammli-Greenberg S (2004). Satisfaction with and access to community care of the chronically ill in Israel’s health system. Health Policy.

[12] Jeon YH, Essue B, Jan S, Wells R, Whitworth JA. Economic hardship associated with managing chronic illness: a qualitative inquiry. BMC Health Serv Res. 2009;9. 10.1186/1472-6963-9-182 PMC2766369 19818128

[13] Goodyear-Smith F, Ashton T (2019). New Zealand health system: universalism struggles with persisting inequities. The Lancet.

[14] Thomson S, Cylus J, Evetovits T. Can people afford to pay for health care? New evidence on financial protection in Europe: World Health Organization. Regional Office for Europe; 2019.

[15] World Health Organization. Health Equity: Social determinants of health: WHOS; 2022 [cited 6 May 2022]. Available from: https://www.who.int/health-topics/social-determinants-of-health#tab=tab_3.

[16] Health and Disability System Review. Health and Disability System Review‐Interim report. Wellington: HDSR 2019.

[17] New Zealand Ministry of Health. Prescription Subsidy Scheme: New Zealand Ministry of Health; 2023 [updated 01 June 2023; cited 24 Nov 2023]. Available from: https://www.health.govt.nz/your-health/conditions-and-treatments/treatments-and-surgery/medications/prescription-subsidy-scheme.

[18] Norris P, Cousins K, Horsburgh S, Keown S, Churchward M, Samaranayaka A (2023). Impact of removing prescription co-payments on the use of costly health services: a pragmatic randomised controlled trial. BMC Health Serv Res.

[19] Manch T. Budget 2023: National vows to bring back $5 prescription fees if elected: Stuff; 2023 [18 May 2023]. Available from: https://www.stuff.co.nz/national/politics/132083751/budget-2023-national-vows-to-bring-back-5-prescription-fees-if-elected.

[20] Patwardhan A, Davis J, Murphy P, Khandelwal N, Sherman B, Manfred J (2011). The impact of 90-day prescriptions on adherence at workplace pharmacies compared to traditional mail order. Popul Health Manag.

[21] Stoneman J, Taylor SJ (2007). Pharmacists’ views on Indigenous health: is there more that can be done?. Rural Remote Health.

[22] Napier P. The introduction of an advanced role for pharmacy technicians into the New Zealand pharmacy setting (Thesis, Doctor of Philosophy) University of Otago: University of Otago; 2017. Available from: http://hdl.handle.net/10523/7702.

[23] Norris P, Tordoff J, McIntosh B, Laxman K, Chang SY, Te Karu L (2016). Impact of prescription charges on people living in poverty: a qualitative study. Res Social Adm Pharm.

[24] Kjoenniksen I, Lindbaek M, Granas AG (2006). Patients’ attitudes towards and experiences of generic drug substitution in Norway. Pharm World Sci.

[25] Gossell-Williams M (2007). Generic substitutions: a 2005 survey of the acceptance and perceptions of physicians in Jamaica. West Indian Med J.

[26] Jones Z. The effect of the increase in prescription charges on Community Pharmacy in New Zealand. [Unpublished report]. 2014.

[27] Chaar B, Brien J, Krass I (2005). Professional ethics in pharmacy: the Australian experience. Int J Pharm Pract.

[28] McDonald S, Lopatka H, Bachynsky J, Kirwin D. Systematic review of pharmacy reimbursement literature. University of Alberta; 1999.

[29] Huttin C (1996). A critical review of the remuneration systems for pharmacists. Health Policy.

[30] Napier P, Norris P, Green J, Braund R (2019). Experiences of pharmacy staff during the introduction of the checking technician role in New Zealand. Int J Pharm Pract.

[31] Khan S (2014). Medicare Part D: pharmacists and formularies—whose job is it to address copays. Consult Pharm.

[32] Norris P, Wilson SE, Green JA, Gu J, Goddard S, Deadman LR (2018). Knowledge and attitudes to prescription charges in New Zealand and England. Res Social Adm Pharm.

[33] Metcalfe S, Beyene K, Urlich J, Jones R, Proffitt C, Harrison J (2018). Te Wero tonu—the challenge continues: Māori access to medicines 2006/07–2012/13 update. N Z Med J.

[34] Yin RK (2018). Case study research and applications.

[35] Aspers P, Corte U (2019). What is qualitative in qualitative research. Qual Sociol.

[36] DeCarlo M. Scientific inquiry in social work 2018 [cited 2021. Available from: https://scientificinquiryinsocialwork.pressbooks.com/chapter/8-4-qualitative-research-questions/.

[37] Jagosh J (2019). Realist synthesis for public health: building an ontologically deep understanding of how programs work, for whom, and in which contexts. Annu Rev Public Health.

[38] Jagosh J (2020). Retroductive theorizing in Pawson and Tilley’s applied scientific realism. J Crit Realism.

[39] Braun V, Clarke V (2021). Thematic analysis: a practical guide.

[40] Alderson P (2021). Critical realism for health and illness research: a practical introduction.

[41] Smith AJ, Scahill SL, Harrison J, Carroll T, Medlicott NJ (2018). Service provision in the wake of a new funding model for community pharmacy. BMC Health Serv Res.

[42] Foster R, Preval N, Blakely T, Wilson N, Desmond James OD. Costing of pharmaceuticals in New Zealand for health economic studies: backgrounder and protocol for costing Technical Report: Number 6. Department of Public Health, University of Otago, Wellington; 2011. Report No.: no.0473198525.

[43] Bay of Plenty District Health Board. Bay of Plenty District Health Board, Hauora A Toi, Annual Report 2021. BOP DHB; 2021.

[44] Qualtrics [Internet]. www.qualtrics.com. 2005 [cited 2023]. Available from: https://www.qualtrics.com/blog/citing-qualtrics/.

[45] Otter [Internet]. 2022. Available from: www.otter.ai.

[46] Wiltshire G, Ronkainen N (2021). A realist approach to thematic analysis: making sense of qualitative data through experiential, inferential and dispositional themes. J Crit Realism.

[47] Fryer T. A critical realist approach to thematic analysis: producing causal explanations. J Crit Realism. 2022:1–20.

[48] Peacocke E.F “It’s my role to ensure that they have access to medicines”: insights from community pharmacists about prescription medicine co-payments and possible implications for equitable access [MPH Dissertation]. Unpublished: University of Otago, New Zealand; 2022.

[49] New Zealand Treasury. Budget 2012 Information Release: T2011/2570: Improving the Targeting of Co-payments in Primary Care: New Zealand Treasury; 2012 [cited 2022 June 27].

[50] Drummond M, Towse A (2012). Is it time to reconsider the role of patient co-payments for pharmaceuticals in Europe?. Eur J Health Econ.

[51] Waitangi Tribunal. Hauora: Report on stage one of the health services and outcomes kaupapa inquiry: Waitangi Tribunal; 2019 [cited 2022 April 10]. Available from: https://waitangitribunal.govt.nz/news/report-on-stage-one-of-health-services-and-outcomes-released/.

[52] Came H, Tudor K (2017). Unravelling the whāriki of Crown Māori health infrastructure. N Z Med J.

[53] New Zealand Cabinet Māori Crown Relations: Te Arawhiti Committee. Treaty of Waitangi guidance (MCR-19-MIN-0024). In: Wellington Cabinet Office, editor. Wellington: Cabinet Office; 2019.

[54] Graham R, Masters-Awatere B (2020). Experiences of Māori of Aotearoa New Zealand’s public health system: a systematic review of two decades of published qualitative research. Aust N Z J Public Health.

[55] Health and Disability System Review. Health and Disability System Review—Final Report—Pūrongo Whakamutunga. Wellington: HDSR; 2020.

[56] Tinirau R, Smith C, Haami M. Whakatika: International Literature Review. Whanganui: Te Atawhai o Te Ao Charitable Trust; 2021.

[57] Durie M (1998). Whaiora: Maōri health development.

[58] World Health Organization. Social Determinants of Health 2021 [cited 1 September 2022]. Available from: https://www.who.int/health-topics/social-determinants-of-health.

[59] Hikaka J, Jones R, Hughes C, Connolly M, Martini N (2021). Ethnic variations in the quality use of medicines in older adults: Māori and Non-Māori in Aotearoa New Zealand. Drugs Aging.

[60] Lee S, Collins FL, Simon-Kumar R (2021). Blurred in translation: the influence of subjectivities and positionalities on the translation of health equity and inclusion policy initiatives in Aotearoa New Zealand. Soc Sci Med.

[61] Reid P, Cormack D, Paine S-J (2019). Colonial histories, racism and health—the experience of Māori and Indigenous peoples. Public Health.

[62] PHARMAC. Achieving medicine access equity in Aotearoa New Zealand: towards a theory of change: PHARMAC; 2019 [Available from: https://pharmac.govt.nz/about/what-we-do/equity/achieving-medicine-access-equity-in-aotearoa-new-zealand-towards-a-theory-of-change/.

[63] Health Quality & Safety Commission New Zealand. Atlas of healthcare variation Wellington: HQSC; 2021 [cited 12 April 2023]. Available from: https://www.hqsc.govt.nz/our-programmes/health-quality-evaluation/projects/atlas-of-healthcare-variation/.

[64] New Zealand Ministry of Health. Key indicators: Results from the 2019/20 New Zealand Health Survey Wellington New Zealand: Ministry of Health; 2021 [updated April 1; cited 14 September 2022]. December 2021: [Available from: https://www.health.govt.nz/publication/annual-update-key-results-2020-21-new-zealand-health-survey.

[65] Weller T, Jamieson C (2004). The expanding role of the antibiotic pharmacist. J Antimicrob Chemother.

